# Adapting Nepal’s polio eradication programme

**DOI:** 10.2471/BLT.16.173674

**Published:** 2016-11-25

**Authors:** Krishna P Paudel, Lee M Hampton, Santosh Gurung, Rajendra Bohara, Indra K Rai, Sameer Anaokar, Rachel D Swift, Stephen Cochi

**Affiliations:** a Government of Nepal Ministry of Health and Population, Kathmandu, Nepal.; b Centers for Disease Control and Prevention, Atlanta, United States of America (USA).; c World Health Organization Expanded Programme on Immunization, Regional Office of the Western Pacific, PO Box 2932, 1000 Manila, Philippines.; d The Boston Consulting Group, Boston, USA.

## Abstract

**Problem:**

Many countries have weak disease surveillance and immunization systems. The elimination of polio creates an opportunity to use staff and assets from the polio eradication programme to control other vaccine-preventable diseases and improve disease surveillance and immunization systems.

**Approach:**

In 2003, the active surveillance system of Nepal’s polio eradication programme began to report on measles and neonatal tetanus cases. Japanese encephalitis and rubella cases were added to the surveillance system in 2004. Staff from the programme aided the development and implementation of government immunization policies, helped launch vaccination campaigns, and trained government staff in reporting practices and vaccine management.

**Local setting:**

Nepal eliminated indigenous polio in 2000, and controlled outbreaks caused by polio importations between 2005 and 2010.

**Relevant changes:**

In 2014, the surveillance activities had expanded to 299 sites, with active surveillance for measles, rubella and neonatal tetanus, including weekly visits from 15 surveillance medical officers. Sentinel surveillance for Japanese encephalitis consisted of 132 sites. Since 2002, staff from the eradication programme have helped to introduce six new vaccines and helped to secure funding from Gavi, the Vaccine Alliance. Staff have also assisted in responding to other health events in the country.

**Lesson learnt:**

By expanding the activities of its polio eradication programme, Nepal has improved its surveillance and immunization systems and increased vaccination coverage of other vaccine-preventable diseases. Continued donor support, a close collaboration with the Expanded Programme on Immunization, and the retention of the polio eradication programme’s skilled workforce were important for this expansion.

## Résumé

**Problème:**

De nombreux pays ont des systèmes de vaccination et de surveillance des maladies déficients. L’élimination de la poliomyélite crée l'occasion d'employer le personnel et les ressources du programme d'éradication de la poliomyélite pour contrôler d'autres maladies évitables par la vaccination et améliorer les systèmes de vaccination et de surveillance des maladies.

**Approche:**

En 2003, le système de surveillance active du programme népalais d'éradication de la poliomyélite a commencé à rendre compte des cas de rougeole et de tétanos néonatal. Les cas d'encéphalite japonaise et de rubéole ont été ajoutés au système de surveillance en 2004. Le personnel du programme a soutenu la création et la mise en œuvre de politiques gouvernementales de vaccination, a aidé à lancer des campagnes de vaccination et a formé du personnel gouvernemental sur les pratiques de signalement et la gestion des vaccins.

**Environnement local:**

Le Népal a éradiqué la poliomyélite autochtone en 2000 et a su contrôler des flambées de poliomyélite importée en 2005 et 2010.

**Changements significatifs:**

En 2014, les activités de surveillance ont été étendues à 299 sites, avec une surveillance active de la rougeole, de la rubéole et du tétanos néonatal comprenant des visites hebdomadaires de 15 médecins de surveillance. En 2014, le réseau de surveillance sentinelle de l'encéphalite japonaise couvrait 132 sites. Depuis 2002, le personnel du programme d'éradication a aidé à introduire six nouveaux vaccins et à pérenniser les financements provenant de Gavi-L'Alliance du vaccin. Ce personnel a également prêté son assistance pour répondre à d'autres épisodes sanitaires dans le pays.

**Leçons tirées:**

En élargissant les activités de son programme d'éradication de la poliomyélite, le Népal a amélioré ses systèmes de surveillance et la couverture vaccinale d'autres maladies évitables par la vaccination. Pour cet élargissement, la pérennisation des aides des donateurs, une étroite collaboration avec le Programme élargi de vaccination et la mobilisation du personnel qualifié du programme d'éradication de la poliomyélite ont joué un rôle clé.

## Resumen

**Situación:**

Muchos países tienen sistemas deficientes de supervisión e inmunización de enfermedades. La eliminación de la poliomielitis ofrece la oportunidad de utilizar el personal y los activos del programa de erradicación de la poliomielitis para controlar otras enfermedades prevenibles mediante vacunación, así como para mejorar los sistemas de supervisión e inmunización de enfermedades.

**Enfoque:**

En 2003, el sistema de supervisión activa del programa de erradicación de la poliomielitis de Nepal comenzó a informar sobre casos de sarampión y tétanos neonatal. En 2004 se añadieron casos de rubéola y encefalitis japonesa al sistema de supervisión. El personal del programa contribuyó al desarrollo y a la implementación de políticas gubernamentales de inmunización, ayudó a lanzar campañas de vacunación y formó a personal público para informar sobre las prácticas y la gestión de las vacunas.

**Marco regional:**

Nepal eliminó la poliomielitis indígena en el año 2000, y controló los brotes provocados por la importación de la poliomielitis entre 2005 y 2010.

**Cambios importantes:**

En 2014, las actividades de supervisión se ampliaron a 299 localizaciones, con supervisión activa del sarampión, la rubéola y el tétanos neonatal, incluidas visitas semanales de 15 médicos de vigilancia. La vigilancia centinela de encefalitis japonesa se realizó en 132 localizaciones. Desde 2002, el personal del programa de erradicación ha contribuido a la introducción de seis nuevas vacunas y ha ayudado a obtener una financiación garantizada por parte de la Gavi, la Vaccine Alliance. El personal también ha ayudado a responder ante otros sucesos sanitarios acaecidos en el país.

**Lecciones aprendidas:**

Mediante la ampliación de las actividades de su programa de erradicación de la poliomielitis, Nepal ha mejorado sus sistemas de supervisión e inmunización y ha aumentado la cobertura de vacunación de otras enfermedades prevenibles mediante vacunación. El continuo apoyo de los donantes, una estrecha colaboración con el Programa Ampliado de Inmunización y la retención del personal cualificado del programa de erradicación de la poliomielitis han sido muy importantes para esta ampliación.

## ملخص

**المشكلة:**

تعاني العديد من البلدان من ضعف نظم رصد الأمراض والتحصين ضدها. ويهيئ القضاء على مرض شلل الأطفال فرصة للاستفادة بالعاملين والمقومات المادية المتاحة من خلال برنامج القضاء على شلل الأطفال للسيطرة على غيره من الأمراض الأخرى الممكن الوقاية منها من خلال التحصينات، وتحسين نظم رصد الأمراض والتحصين ضدها.

**الأسلوب:**

في عام 2003، بدأ نظام الرصد النشط الخاص ببرنامج القضاء على شلل الأطفال في نيبال الإبلاغ عن حالات الإصابة بالحصبة والتيتانوس بين الأطفال حديثي الولادة. وتمت إضافة حالات مرض التهاب الدماغ الياباني والحصبة الألمانية إلى نظام الرصد في عام 2004. وساعد العاملون في البرنامج في تطوير وتنفيذ سياسات التحصين الحكومية، كما ساعدوا في طرح حملات التحصين، وساعدوا في تدريب العاملين بالحكومة في الإبلاغ عن الإجراءات المتبعة وإدارة التحصين.

**المواقع المحلية:**

قضت نيبال على الحالات المستوطنة لديها من شلل الأطفال في عام 2000، وسيطرت على الحالات المتفشية الناتجة عن إصابات شلل الأطفال الواردة من الخارج في الفترة ما بين 2005 و2010.

**التغيّرات ذات الصلة:**

في عام 2014، توسعت أنشطة الرصد لتشمل 299 موقعًا، مع بذل جهود نشطة لرصد الحصبة العادية والحصبة الألمانية والتيتانوس بين الأطفال حديثي الولادة، بما في ذلك الزيارات الأسبوعية من 15 مختصًا طبيًا لرصد الأمراض. ومنذ عام 2002، ساعد العاملون في برنامج القضاء على الأمراض في طرح ستة تحصينات جديدة، كما ساعدوا على الحصول على تمويل من التحالف العالمي للقاحات والتحصين. كما ساعد العاملون في الاستجابة للوقائع الصحية الأخرى في البلاد.

**الدروس المستفادة:**

من خلال توسيع نطاق أنشطة برنامج القضاء على مرض شلل الأطفال، فقد حسّنت نيبال من نظم الرصد والتحصين لديها، كما زادت من تغطية التحصين ضد الأمراض الأخرى القابلة للوقاية منها بالتحصينات. ومع استمرار دعم الجهات المانحة، فقد كان من المهم إجراء التعاون في إطار وثيق مع برنامج التحصين الموسّع، والحفاظ على فريق العمل المهاري لبرنامج القضاء على شلل الأطفال بغرض توسيع نطاق البرنامج.

## 摘要

**问题:**

许多国家的疾病监测和免疫接种系统都很薄弱。 根除脊髓灰质炎项目为利用根除脊髓灰质炎项目工作人员和资产控制其他疫苗可预防疾病并完善疾病监测和免疫接种系统创造了机会。

**方法:**

在 2003 年，尼泊尔根除脊髓灰质炎项目主动监测系统开始就麻疹及新生儿破伤风病例进行报告。 2004 年，该监测系统中增添了日本的脑炎和风疹病例。项目工作人员为政府免疫接种政策的制定与实施提供援助，帮助启动疫苗接种活动并为政府工作人员提供实践报告和疾病管理方面的培训。

**当地状况:**

尼泊尔于 2000 年在全国根除了脊髓灰质炎并在 2005 年至 2010 年间控制了由于脊髓灰质炎病例输入引起的疾病爆发。

**相关变化:**

2014 年，监测活动扩展到了 299 个地区，对麻疹、风疹和新生儿破伤风病例进行主动监测，包括由 15 名监测医疗人员进行每周随访。 对日本脑炎病例的哨点监测涵盖 132 个地区。 自 2002 年以来，根除项目工作人员已帮助引进 6 种新疫苗并获得全球疫苗免疫联盟 (Gavi) 的资助。 工作人员还协助应对国内发生的其他卫生事件。

**经验教训:**

通过扩展根除脊髓灰质炎项目的活动范围，尼泊尔改善了其监测和免疫接种系统，并增加了其他疫苗可预防疾病的疫苗接种覆盖率。 持续的捐助者支持、与扩大免疫规划 (Expanded Programme on Immunization) 的密切配合以及对根除脊髓灰质炎项目技能熟练劳动力的保留对于扩大该项目非常重要。

## Резюме

**Проблема:**

Во многих странах слабо развиты системы наблюдения за заболеваниями и иммунизации. Искоренение полиомиелита позволит использовать персонал и ресурсы программы по искоренению полиомиелита для борьбы с другими заболеваниями, предотвращаемыми с помощью вакцинации, и улучшить системы наблюдения за заболеваниями и иммунизации.

**Подход:**

В 2003 году система активного наблюдения программы по искоренению полиомиелита начала отчитываться о случаях заболевания корью и неонатальным столбняком. В 2004 году наблюдение было распространено на японский энцефалит и краснуху. Персонал, участвующий в этой программе, содействовал развитию и внедрению правительственных политик по иммунизации, помогал развернуть кампании по вакцинации и обучал государственных служащих процедурам отчетности и обращению с вакцинами.

**Местные условия:**

Эндемический полиомиелит в Непале был искоренен в 2000 году, а в период между 2005 и 2010 годами велась борьба с полиомиелитом, занесенным извне.

**Осуществленные перемены:**

В 2014 году эпидемиологический надзор был расширен до 299 пунктов, в которых осуществлялось активное наблюдение за корью, краснухой, неонатальным столбняком, включающее еженедельные визиты 15 медработников системы наблюдения. Дозорный эпиднадзор за японским энцефалитом осуществлялся силами 132 пунктов. С 2002 года персонал программы по искоренению полиомиелита помог внедрить шесть новых вакцин и обеспечить финансирование со стороны Глобального альянса по проблемам вакцинации и иммунизации (GAVI). Персонал также участвовал в реагировании на другие события в области здравоохранения в стране.

**Выводы:**

Расширение деятельности в рамках программы по искоренению полиомиелита позволило улучшить непальские системы наблюдения и иммунизации и увеличить охват вакцинацией других заболеваний, предотвращаемых с помощью вакцинации. Постоянная поддержка доноров, тесное сотрудничество с расширенной программой иммунизации и удержание высококвалифицированного персонала программы по искоренению полиомиелита играли важную роль в этом расширении.

## Introduction

National immunization systems are important for reducing vaccine preventable diseases.^1^ However, in many resource-constrained countries, such systems need to be improved. This paper describes how Nepal’s polio eradication programme expanded its work to aid efforts to control other vaccine- diseases and improve Nepal’s disease surveillance and immunization systems.

## Local setting

Nepal eliminated indigenous polio in 2000 and controlled outbreaks caused by polio importations between 2005 and 2010. The country participated in the certification of wild poliovirus elimination in the World Health Organization (WHO) South-East Asia Region in 2014.^2^

Nepal’s polio eradication programme, created in 1998, is funded by the Global Polio Eradication Initiative and is affiliated with WHO’s Nepal country office. The original aim of the programme was to conduct active surveillance for possible polio cases and to provide technical assistance and support on polio vaccination to the country’s Expanded Programme on Immunization (EPI), which is the national immunization programme. In 2002, the polio eradication programme had 14 field-office based surveillance medical officers, who actively searched for people with acute flaccid paralysis (AFP), i.e. suspected polio cases.^3^ The programme used these surveillance data to guide polio immunization activities, especially mass campaigns with oral poliovirus vaccine.

Nepal’s EPI began as a pilot project in three districts in 1979, and by 1988 had expanded into a nationwide immunization system providing Bacillus Calmette–Guérin, diphtheria-tetanus-pertussis, polio, tetanus and measles vaccines.^4^^,^^5^ In 2000, EPI was obtaining information on vaccine-preventable disease cases, except polio, mainly from Nepal’s health management information system. This information system collects reports of infectious disease cases from all government health facilities in Nepal,^4^^,^^5^ but like many other passive surveillance systems, it has problems with underreporting, delayed reporting or incomplete reporting.^4^^,^^5^

## Approach

The elimination of indigenous polio created an opportunity to use resources from the polio eradication programme to strengthen the efforts to control other vaccine-preventable diseases.

In 2003, the polio eradication programme’s AFP surveillance system began collecting data on measles and neonatal tetanus cases.^4^^,^^5^ The data collection initially involved weekly reports to the polio eradication programme from the staff at 413 hospitals and major health facilities, including all inpatient hospitals, located throughout the country. Surveillance medical officers made weekly visits to 84 active surveillance sites among the 413 health facilities to review records of illnesses treated at those facilities and to interview health facility staff regarding possible new preventable disease cases. During their visits, they also compared their findings to the contents of the weekly reports, helped to ensure that blood and stool samples were collected and sent to the appropriate laboratories, and trained and motivated health workers regarding the identification, documentation, and reporting of new preventable disease cases. Blood samples were collected for measles confirmatory testing during investigations of suspected measles outbreaks. In 2004, the surveillance system expanded to include rubella, acute encephalitis syndrome and Japanese encephalitis.^5^^–^^7^ Due to the similarity in clinical presentation between rubella and measles, surveillance for rubella began by testing for rubella immunoglobulin (Ig) M in blood samples from suspected measles cases that tested negative for measles IgM.^5^ To detect acute encephalitis syndrome cases, a system of 45 sentinel medical facilities, located primarily in districts suspected to have the highest Japanese encephalitis risk, was established through a joint effort of the polio eradication programme, the Government of Nepal and several domestic and international laboratories and technical agencies. This sentinel system used the same database and reverse cold chain for shipping laboratory samples as the AFP surveillance system. Laboratory testing of blood or cerebrospinal fluid samples confirmed Japanese encephalitis cases.^6^^,^^7^

Since 2002, the staff from the eradication programme have used their experience to help improve multiple aspects of EPI. They have provided continuous training and support to EPI staff on issues such as vaccine cold chain management, data management, and assessment of adverse events. They aided the development and implementation of policy guidelines. They have also assisted in supervising and monitoring routine immunization activities; assisted in microplanning to reach every district; enhanced research and grant writing capabilities; and piloted innovations, such as electronic immunization records and immunization training centres.

## Relevant changes

### Surveillance

**Fig. 1:**
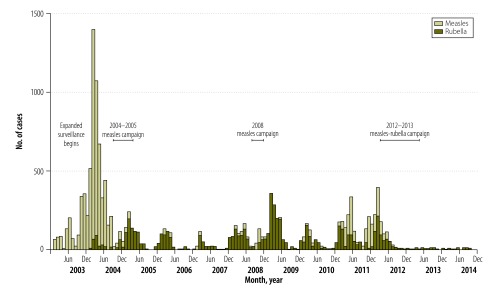
Confirmed measles and rubella cases in Nepal, 2003–2014

Note: Confirmed measles and rubella cases are suspected measles cases (generalized maculopapular rash and fever plus one of the following: cough, coryza or conjunctivitis) with either laboratory confirmation through measles or rubella immunoglobulin M testing or an epidemiological link to a measles or rubella outbreak with a laboratory-confirmed case.

In 2014, the surveillance system activities had expanded to 299 sites, with active surveillance for measles, rubella and neonatal tetanus, including weekly visits from 15 surveillance medical officers. The sentinel system for acute encephalitis syndrome consisted of 132 sites. Information from the expanded surveillance has helped to guide the development and implementation of immunization policies. For example, in 2003, the surveillance system detected 1536 confirmed measles cases, which indicated the need of a vaccination campaign for people younger than 15 years (Fig. 1).^5^ EPI acted on this information and with the help of the eradication programme launched a measles vaccination campaign in 2004–2005. Following the campaign, 45 confirmed measles cases were identified in 2006.^5^ In 2008, an increase in measles cases, a total of 394 confirmed cases, prompted a follow-up vaccination campaign in 2008, targeting children aged 9 months to 4 years.^8^ The detection of additional measles cases in 2011, along with several years of data indicating a substantial burden of rubella, led to a combined measles–rubella vaccination campaign in 2012–2013 targeting all children between the ages of 9 months and 14 years. Afterwards, EPI introduced rubella vaccine into the country’s routine immunization schedule. Subsequently, the numbers of confirmed measles and rubella cases have fallen, with only 19 measles and 37 rubella cases in 2013 and 2014 combined. However, further efforts are needed to achieve the goal of eliminating measles in Nepal.^8^

Data source: Measles and rubella surveillance, World Health Organization Programme for Immunization Preventable Diseases, Nepal.

In 2005, the data from the surveillance system confirmed the finding from the health management information system that Nepal had met the criteria for the elimination of neonatal tetanus (< 1 case of neonatal tetanus per 1000 live births in every district).^4^ The surveillance system has continued to monitor for any increase in neonatal tetanus cases.

The data from the surveillance system also aided the planning of Nepal’s first Japanese encephalitis vaccination campaign in 2006, by identifying the high-risk districts.^6^ After the campaign, surveillance data demonstrated an 84% drop (from 864 to 141) in Japanese encephalitis cases in these districts.^7^ By 2011, EPI had completed Japanese encephalitis vaccination campaigns in 31 high- and moderate-risk districts and they had introduced the vaccine into routine immunization in those districts. In 2016, EPI included nationwide Japanese encephalitis vaccination in the routine immunization schedule.

### Immunization system strengthening

Since 2002, staff from the eradication programme have helped EPI to introduce hepatitis B vaccine (2003), *Haemophilus influenzae* type b vaccine (2009), inactivated polio vaccine (2014) and pneumococcal conjugate vaccine (2015), in addition to rubella vaccine and Japanese encephalitis vaccine. For example, for the introduction of inactivated polio vaccine, staff from both programmes worked together on an application for support from Gavi, the Vaccine Alliance. They planned together how to roll out the vaccine, develop training materials, and supervise and monitor the introduction of the vaccine. In 2014, Nepal became the first country, among those receiving support from Gavi, to introduce this vaccine.^9^ They also planned, organized and executed the replacement of trivalent inactivated oral polio vaccine with bivalent inactivated vaccine on 17 April 2016. By monitoring the replacement, they could confirm by 11 May 2016 that all trivalent oral polio vaccine had been removed from the country’s vaccine storage sites and health facilities providing immunizations.

The staff from the eradication programme have assisted in the development of the *Comprehensive multiyear plan 2068–2072 (2011–2016)*^10^ and the national plan of action on intensification of routine immunization.

Between 2001 and 2015, the eradication programme’s work contributed to the increase in vaccination coverage among Nepalese children aged 12–23 months. WHO and the United Nations Children’s Fund estimated that the proportion of children who received three doses of diphtheria-pertussis-tetanus containing vaccine rose from 72% to 91% and the estimated proportion who received one dose of measles vaccine rose from 71% to 85%.^11^

The staff from the eradication programme have also assisted in responding to other health events, such as dengue fever and cholera outbreaks and natural disasters. Following the major earthquake and subsequent aftershocks in April 2015, the staff assisted in monitoring for potential disease outbreaks, assessed damage to health facilities and helped identify needs for disaster relief.^12^

## Discussion

The polio eradication programme in Nepal has transitioned from focusing solely on polio to working on preventing other vaccine-preventable diseases. This transition has shown how staff and assets from an eradication programme can both strengthen a country’s immunization system and reduce disease incidence (Box 1). Several factors contributed to this successful transition, including the eradication programme’s ability to collaborate with EPI, international technical agencies and donors; the level of support received from donors for the expanded activities; and the eradication programme’s well-trained, highly capable and motivated staff. Nepal’s immunization system has benefited from external assistance. For example, Gavi has funded 60–70% of the costs of vaccine purchases in the country. The total vaccine cost in 2014 was approximately 6.5 million United States dollars. The immunization system has also been strengthened by the January 2016 enactment of the national immunization law that requires that the government allocate adequate funding for immunizations and establishes a fund for collecting national private sector donations for support of EPI.^13^

Box 1 **Summary of main lessons learnt**

- Programmes created specifically to eliminate polio can successfully expand their efforts to strengthen immunization systems’ abilities to address other vaccine preventable diseases.
- Expanding polio surveillance systems to detect other vaccine-preventable diseases can effectively guide subsequent efforts to improve immunization systems.
- The elimination of polio in a country provides an opportunity to transition polio eradication staff, assets and experiences to other projects.

Other countries have also enlisted their polio eradication programmes in their work on other diseases. For example, in 2007, Bangladesh and India initiated surveillance of Japanese encephalitis with the aid of polio surveillance officers.^14^ In Nigeria, polio staff and their organizational experience were used to quickly end an outbreak of Ebola virus disease in 2014.^15^ The possibility that polio may be eradicated in the next few years suggests that staff and assets currently funded by the Global Polio Eradication Initiative may increasingly become available in other areas. Countries should carefully manage the transition from polio eradication to other immunization and public health priorities to ensure that they effectively use valuable experience and assets from the Global Polio Eradication Initiative after the initiative ends.^16^
